# An Atlas of Thyroid Hormone Receptors’ Target Genes in Mouse Tissues

**DOI:** 10.3390/ijms231911444

**Published:** 2022-09-28

**Authors:** Yanis Zekri, Romain Guyot, Frédéric Flamant

**Affiliations:** Institut de Génomique Fonctionnelle de Lyon, University Lyon, CNRS UMR 5242, INRAE USC 1370 École Normale Supérieure de Lyon, Université Claude Bernard Lyon 1, 46 Allee d’Italie, 69364 Lyon, France

**Keywords:** thyroid hormone receptors, RNA-seq, ChIP-seq, atlas, target genes

## Abstract

We gathered available RNA-seq and ChIP-seq data in a single database to better characterize the target genes of thyroid hormone receptors in several cell types. This database can serve as a resource to analyze the mode of action of thyroid hormone (T3). Additionally, it is an easy-to-use and convenient tool to obtain information on specific genes regarding T3 regulation or to extract large gene lists of interest according to the users’ criteria. Overall, this atlas is a unique compilation of recent sequencing data focusing on T3, its receptors, modes of action, targets and roles, which may benefit researchers within the field. A preliminary analysis indicates extensive variations in the repertoire of target genes where transcription is upregulated by chromatin-bound nuclear receptors. Although it has a major influence, chromatin accessibility is not the only parameter that determines the cellular selectivity of the hormonal response.

## 1. Introduction

Thyroid hormone (3,3′,5-triiodo-L-thyronine or T3) exerts a broad influence on vertebrate development and adult physiology. It is notably known to trigger the metamorphosis of frogs and fish [1]. During mammalian development, it is required for proper brain maturation and bone growth and becomes a main regulator of energy metabolism in adults. At the cellular level, T3 often stimulates differentiation [2,3,4,5] and mitochondrial metabolism [6].

T3 exerts its influence by binding to the nuclear receptors TRα1, TRβ1 and TRβ2 (collectively TR) encoded by the two genes *Thra* and *Thrb* [7]. TR act as heterodimers with other nuclear receptors, mainly RXR. They bind to specific thyroid response elements (TREs) constituted by doublets of the AGGTCA half-site (DR4 elements) [8] present in the regulatory sequences of genes, where transcription is upregulated on T3 binding [9]. Although both *Thra* and *Thrb* genes have distinct expression patterns, all cell types express at least one of the two genes and are thus potentially T3 responsive. However, it seems that T3 activates the transcription of various genes in different cell types, which explains why it exerts different physiological influences depending on the tissue. The molecular basis for the cell type specificity of the T3 response is currently unclear.

Due to the broad influence of T3 on gene expression in many cell types, an inventory of the TR target genes should enlighten us on many important developmental and physiological functions. This represents, however, a tremendous task. A previous compilation of genome-wide expression analyses, mainly based on microarray analyses, identified only a few genes, which were regulated by T3 in several cell types or tissues [10]. Since this early attempt, several novel studies have been performed on mice. Both RNA-seq and ChIP-seq analyses have been used to better characterize TR target genes in several cell types or tissues. We present here an atlas of the currently available datasets, which can be used as a novel resource to explore the many functions of T3 and from which we try to extract general conclusions on T3 signaling.

## 2. Results

### 2.1. Definition of TR Target Genes

T3 alters gene expression in exposed cells both directly, by binding the chromatin-bound TR, and indirectly, because TR target genes can encode transcription regulators, which rapidly generate a secondary response. Unraveling these two overlapping processes is difficult, although this can be done in vitro with a translation inhibitor [11,12,13]. Here, we will use two simple criteria to define TR target genes:

(1) Genes that are upregulated after T3 treatment of hypothyroid mice. Initial T3 depletion is important because some genes are more sensitive to it than to an excess of T3 [14]. Hypothyroidism is obtained most of the time by pharmacological means with at least two weeks of treatment with propyl-thio-uracyl (PTU) or methimazole (MMI). In vitro, serum used for cell cultures should be depleted of T3. When a time-course analysis is performed, a rapid response, within hours, is a good indication that the transcriptional activation is a direct consequence of TR-mediated regulation;
(2) Regulatory sequences are occupied by either TRα1 or TRβ1/2, as evidenced by immunoprecipitation of chromatin. Until now, the difficulty of raising high-quality antibodies against TR, which are not abundant proteins, has limited the production of ChIP-seq datasets for the liver [15,16]. This technical limitation is now commonly circumvented by using tagged receptors, for which several transgenic mice have been produced [17,18]. The availability of a ‘floxed’ construct [19] allows us to address chromatin occupancy by TRα1 in a single cell type within a heterogeneous tissue.

### 2.2. Presentation of the Atlas

Table 1 shows all mouse datasets that are currently available in the literature to our knowledge, linking transcriptome (RNA-seq) and cistrome (ChIP-seq) data to facilitate the identification of TR target genes. We excluded data obtained on cell lines, and included data obtained from primary cell cultures. Overall, we collected eight RNA-seq datasets accounting for different brain and non-brain tissues, covering cell types of very different functions and embryonic origins. It is worth noting that these datasets emerge from different protocols, using: (1) mice of different ages, (2) submitted to different hypothyroidism procedures or no procedures, (3) treated for different periods and with different doses of thyroid hormone and (4) with thyroid hormone administrated in different manners. These differences undoubtedly trigger responses of very different magnitudes, making quantitative comparisons between tissues difficult.

We also collected seven ChIP-seq datasets, including two for GABAergic neurons at different developmental stages and three for the liver. Some of these datasets display different thyroid statuses, allowing us to study the consequences of T3 binding to TRs on chromatin occupancy.

To avoid bias, we reanalyzed all datasets using a homogeneous analytical pipeline for sequence mapping and counting of gene coverage. Further details are presented in the Methods section. Finally, all these data were combined into a single atlas (Table S1), where one can find expression and chromatin-occupancy data for each gene. A tutorial has been added to the atlas in a dedicated worksheet to help the reader with its use.

### 2.3. Differentially Expressed Genes

**Figure 1:**
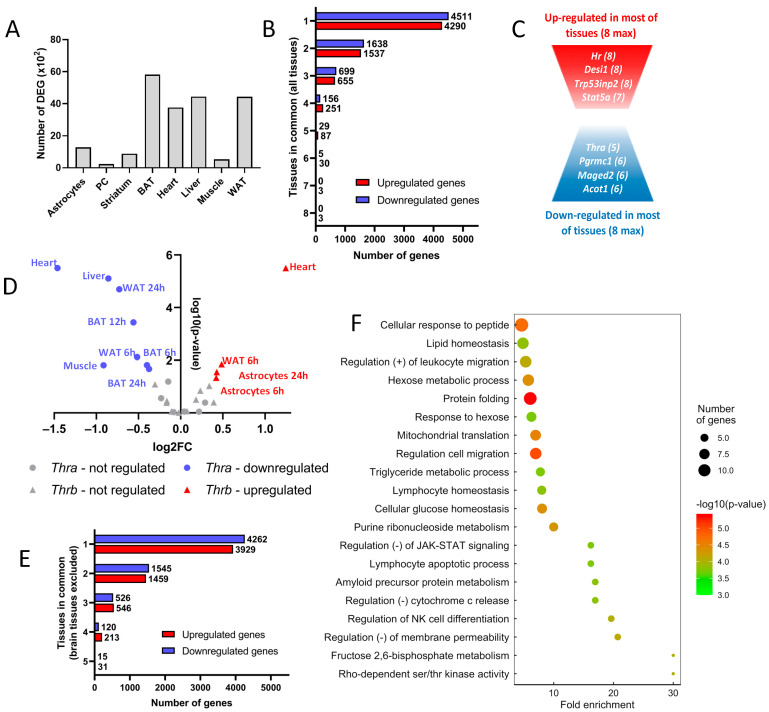
Thyroid hormone regulates the expression of tissue-specific genes. (**A**) Number of differentially expressed genes (DEG, ×10^2^) in available tissues. (**B**) Number of genes up- or downregulated shared between tissues. Genes were classified into eight categories, depending on the number of tissues within which they were regulated. (**C**) Schematic representation of the up- and downregulated genes shared among tissues. (**D**) Regulation of *Thra* (circle) and *Thrb* (triangle) by thyroid hormone. Log_2_ fold-change and statistical significance were plotted for each time point of each tissue. When the regulation was significant (that is, when the −log_10_ *p*-value > 1.3, which means the *p*-value < 0.05), tissues within which *Thra* or *Thrb* were up- or downregulated were colored red and blue, respectively. The name of the tissue and the time point of the kinetics were attributed to each point. (**E**) Number of genes up- or downregulated among tissues, excluding brain tissues. Genes were classified into five categories, depending on the number of tissues within which they were regulated. (**F**) Gene ontology dot plot of the genes upregulated in at least four of the five non-brain tissues. Some of the terms were shortened to increase the readability without affecting the meaning.

Differential gene expression analysis identified thousands of genes where the expression level is modified, either in vivo or in vitro, after T3 treatment (Table S1). The number of differentially expressed genes (DEGs) varies extensively from one cell population to the next (Figure 1A). This suggests that different cell types display different sensitivities to T3. According to Table S1, this feature cannot simply be explained by variations in the expression of *Thra* and *Thrb*. It is worth noting that the different protocols used may also explain this difference. In particular, as mice were not made hypothyroid prior to T3 treatment, the effects on the muscle transcriptome are less visible.

The vast majority of DEGs are not shared by the different cell types (Figure 1B). To the well-known *Hr* gene [24], which encodes the HAIRLESS transcription cofactor, one can add *Desi1, Trp53ind* and *Stat5a* as genes upregulated in most, if not all, cell types (Figure 1C). *Stat5a*, which encodes a member of the STAT family of transcription factors, raises interesting possibilities for cross-talk between T3 signaling and other signaling pathways, which remain to be explored. While no genes were repressed by T3 in all cell types, we still identified a set of genes with this recurrent pattern. Interestingly, it included *Thra* itself, which might provide a molecular basis for a negative feedback loop. Therefore, we looked more precisely at *Thra* and *Thrb* regulation and observed that *Thrb* had the opposite trend (Figure 1D), although not as pronounced as in metamorphosing amphibians [25].

Since the T3 response of cells outside the brain is thought to be mainly metabolic, while neural cells’ T3 response is described as mainly relevant to terminal differentiation and maturation, we analyzed the five datasets separately, excluding brain tissues. Although they shared a higher fraction of DEG (Figure 1E), the overlap between the sets of DEG remained limited. We used gene ontology to identify biological functions enriched in these cell types and found a significant enrichment for several gene sets (Figure 1F). While the categories ‘lipid homeostasis’ and ‘mitochondria translation’ were expected, other elements were more surprising, notably those suggesting immune infiltration. In general, this analysis indicates that different cell types show very different responses to T3, with some similarities when only the responses of cell types outside the brain are considered.

### 2.4. Chromatin Occupancy by TR

**Figure 2:**
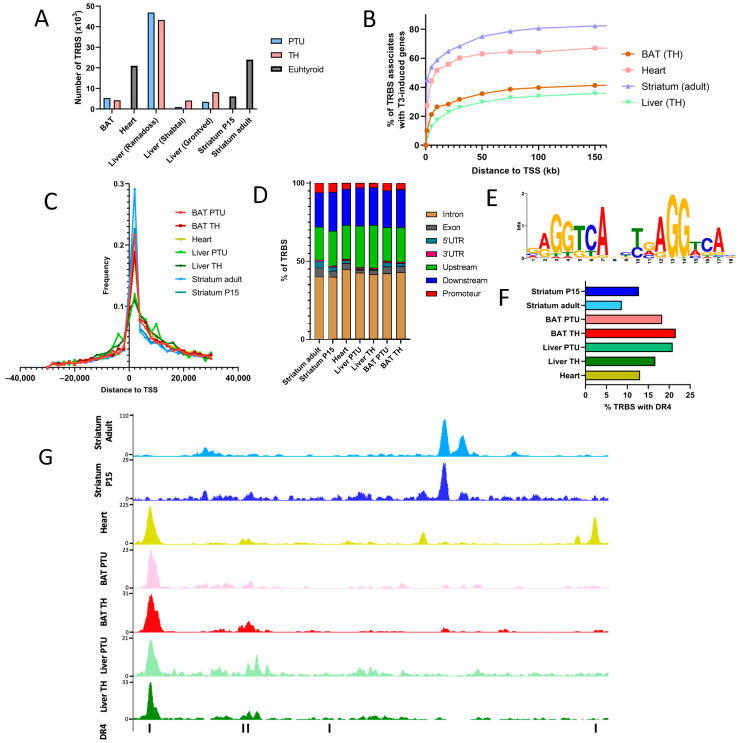
TR chromatin occupancy. (**A**) Number of TR binding sites (TRBS) in each of the available tissues. The different thyroid statuses are indicated by a color code (blue: hypothyroid by exposure to PTU, pink: TH injection, grey: euthyroid). (**B**) Percentage of genes upregulated by T3 that possess a TRBS depending on the distance (in kilobase) chosen to attribute a TRBS to a gene. We generally observed three phases: a very high enrichment of T3 induced when considering TRBS within 10 kb, a high enrichment for TRBS within 30 kb and a plateau above 50 kb. Thus, we chose 30 kb as a distance to attribute a TRBS to a gene, as a compromise to maximize the number of TR target genes while minimizing false positives. (**C**) Frequency of TRBS relative to their distance from the transcription starting site (TSS). (**D**) Distribution of TRBS in different gene regions. (**E**) Consensus sequence identified by a de novo motif search in brown adipocytes. All available tissues display a similar DR4 element to the top enriched motif. (**F**) Percentage of TRBS that possess a DR4. (**G**) Extract of the Integrative Genome Viewer (IGV) in the promoting region of *Hcn2*. Each tissue is represented by a different color, and DR4 elements are represented by black vertical lines at the bottom of the IGV shot.

The number of TR binding sites (TRBS) in the chromatin identified by ChIP-seq analysis is highly variable (Figure 2A), which might reflect either technical variations or a genuine influence of the cellular environment on chromatin occupancy. The fact that three studies address TR occupancy in the liver, in which the T3 response is almost exclusively mediated by TRβ1, allows us to identify the technical sources of this variability. The overexpression of tagged TRβ1 by an adenovirus vector [23] greatly helps in the detection of chromatin occupancy sites, but probably facilitates the binding of the receptor to chromatin. In particular, it erases the influence of T3 on chromatin accessibility, which is observed in the two other liver studies. In addition, it drastically increases the mean number of peaks within the same gene. In the following, we will favor the study that uses transgenic mice with a tagged TRβ1 [18] because it takes advantage of the tagging of TRβ1 to produce high-quality data without taking the risk of generating artificial occupancy by overexpression.

The common way to combine transcriptome and cistrome data is to set an arbitrary distance between the TRBS and the transcription start site (TSS). The gene is then called a TR target gene if its expression is T3 responsive and if a TRBS is present within the interval given by this threshold distance. Different studies vary in the definition of this distance, which results from a compromise: a long distance will produce more false positives, while a small distance will increase the rate of false negatives. For our novel analysis, we calculated the fraction of T3-responsive genes that possess a TRBS depending on the distance choice (Figure 2B). We concluded that a 30 kb distance was a good compromise. Intriguingly, the distribution of the TRBS with respect to the TSS is not symmetrical, and the fact that they are more frequent downstream to the TSS is, at first sight, counterintuitive (Figure 2C). Accordingly, a large fraction of the TRBS is found in introns (Figure 2D).

ChIP-seq does not only capture the direct association of proteins to DNA but also the indirect connections mediated by protein–protein interactions, also called “protein tethering”. Motif analysis can be viewed as a way to better identify all the binding proteins and also to define consensus sequences for each protein that binds to chromatin. We performed a de novo motif search in each dataset and the results were remarkably similar. A single consensus sequence was detected, which corresponds to the so-called DR4 element (Figure 2E). The association of the TRα1/RXRα heterodimer with this element has previously been analyzed in great detail by X-ray crystallography [26]. TR occupies the downstream half-site, while RXR binds to the 5′ half-site. The sequence of the four nucleotides’ spacer is less precisely defined, but the last two nucleotides also establish contact with TR. Depending on the statistical threshold for similarity, the detection of the consensus sequence in TRBS varies. Using a file compiling all the DR4 in the mouse genome (Table S2), we found DR4 elements in only 10–25% of the identified TRBS (Figure 2F), which leaves open the possibility for other modes of association of TR with chromatin.

Thus, DR4-like elements occupied by TR represent only a few thousand of the 70,394 DR4-like elements present in the mouse genome. This highlights that chromatin accessibility is an important factor, which governs the occupancy of DR4 elements by TR. Accordingly, within the same genomic location, we can observe very different patterns (Figure 2G). Some TRBS will be detected only in non-brain tissues, with the underlying absence of a DR4 site. Some TRBS will be unique, with or without the presence of a DR4, while some DR4 will be present in unoccupied sites.

### 2.5. Evolution of Chromatin Occupancy during Cell Differentiation and after T3 Treatment

**Figure 3:**
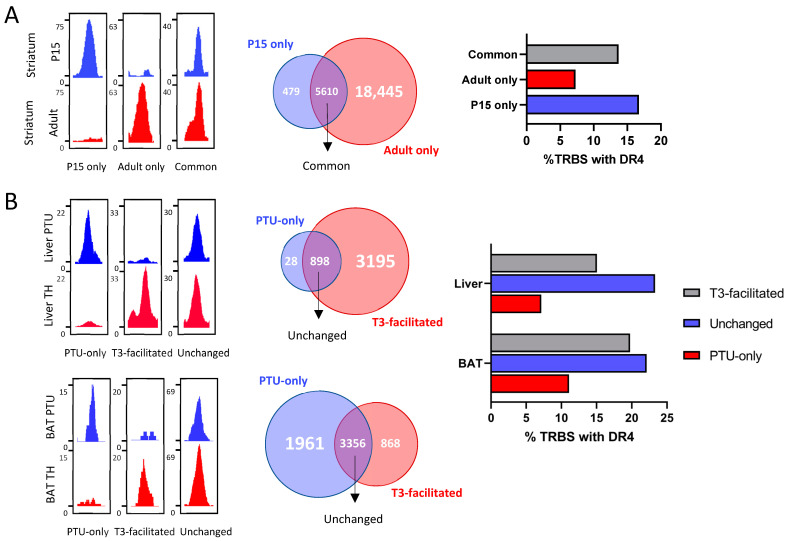
Plasticity of TR during development and T3 treatment. (**A**) *(Left)* Integrative Genome Viewer shots of TRBS categorized as only appearing in P15 striatum, adult striatum or present in both. *(Middle)* Venn diagram of TRBS present on each of the defined categories. *(Right)* Percentage of TRBS that possess a DR4 element. (**B**) (*Left)* Integrative Genome Viewer shots of TRBS categorized as only present in the PTU condition, facilitated by T3 or present independently of T3 status. *(Middle)* Venn diagram of TRBS present on each of the defined categories. *(Right)* Percentage of TRBS that possess a DR4 element. This analysis was carried out for the liver *(top)* and brown adipocytes *(bottom)*.

The previous analysis outlines striking differences in chromatin occupancy among cell types. The available ChIP-seq datasets allow us to better understand the source of these variations. In particular, the analysis of chromatin occupancy in GABAergic neurons of the striatum has been performed using the same protocol at two different stages of life: juvenile, when neuronal circuits are still immature, and adult. The number of TRBS is much higher in adults, suggesting better accessibility of putative binding sites. However, this is not the only parameter that evolves with time, because some TRBS are also lost during striatum maturation (Figure 3A). The fact that TRBS-containing DR4 represents a smaller fraction among TRBS gained in adulthood raises an interesting possibility: it could reflect the assembly of multiprotein complexes containing TRα1. These would contact chromatin at several different points and thus produce several TRBS. In fact, it is frequently observed that T3-responsive genes often contain more than one TRBS, among which a single DR4 element is present.

We also address the influence of T3 on chromatin occupancy, a question that was previously investigated in depth in liver studies [18,21]. In the liver, T3 increases the number of TRBS, and this is not due to an induction of *Thrb* expression. Again, some TRBS are lost, while others are gained soon after T3 treatment (Figure 3B). The enlargement of the cistrome after T3 treatment is unlikely to become a general rule as an opposite trend is found in brown adipocytes, although this conclusion relies on a single dataset.

### 2.6. Combination of Transcriptome and Cistrome Data

**Figure 4:**
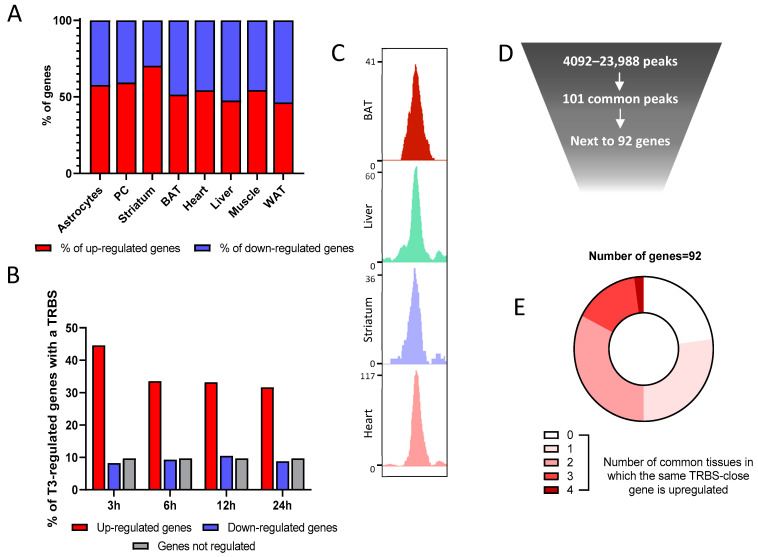
Combined analysis of RNA-seq and ChIP-seq data gives insights into TR mechanisms of action. (**A**) Percentage of genes up- or downregulated by thyroid hormone in the different available tissues. (**B**) Percentage of genes that possess a TRBS within 30 kb of their TSS among genes where the expression in the BAT is either regulated or not by T3 (upregulated in red, downregulated in blue and a set of randomly selected nonregulated genes in grey). (**C**) Integrative Genome Viewer shot of a TRBS shared in the four different available tissues. (**D**) Schematic representation of the rarity of common TRBS. (**E**) Circle diagram of the T3 sensitivity for the 92 genes that are close to the 101 common TRBS. These genes are divided into five categories, decided by in how many of the five tissues their expression is induced by T3.

RNA-seq indicates that an equivalent number of genes are downregulated or upregulated after T3 treatment (Figure 4A), which might lead researchers to question the idea that liganded TR are only transcription activators. Nonetheless, 45% of genes upregulated in the BAT after 3 h of T3 possess a TRBS within 30 kb of the TSS, a proportion that falls to 35% over time, certainly due to the accumulation of indirect regulations. However, this is the case for less than 10% of downregulated genes, a proportion similar to genes insensitive to T3 (Figure 4B). This suggests that the negative regulation of gene expression is not directly mediated by liganded TR and could be secondary, for example, to the upregulation of genes encoding transcription repressors.

A large fraction of the TRBS was not located next to T3 responsive genes, which can garner three nonexclusive explanations. (1) First, it could be the result of chromatin folding that enables TR to act at a very long distance, as other nuclear receptors do [27]. (2) Another possibility is that a large fraction of TRBS is not functional, reflecting a mode of association of TR with DNA that does not allow for the recruitment of transcription coactivators. This could eventually result from the labile interaction of TR with low-affinity DNA-binding motifs, as recently suggested by in vitro assays [28]. (3) Finally, the cellular context may be determinant, and cell-specific coactivators may be involved in converting T3 binding to chromatin-associated TR into transcription activation. To better consider this last possibility, we focused the analysis on the TRBS shared by the four available tissues (Figure 4C). These 101 shared TRBS (Figure 4D) are located next to 92 genes, and 40% of them contain DR4 elements, a ratio above the average frequency. However, only two of these genes are T3 responsive in the analyzed cell types (Figure 4E). This evidences that TR binding is necessary but not sufficient for T3 transactivation, and that cell-specific parameters are involved in converting TR binding to transcriptional activation.

## 3. Discussion

The present study addresses the mechanisms of T3-mediated transactivation by gathering and analyzing in a uniform manner the currently available datasets. This article outlines how different cell types respond to T3 stimulation in very different ways. The overlap between the repertoires of TR target genes in different cell types remains limited, even for cell types in which T3 activates energy metabolism and mitochondrial functions, such as cardiomyocytes, hepatocytes and adipocytes.

We can speculate that this variability also occurs within each tissue. Indeed, the T3 response analyzed at the whole organ level results from the combination of the T3 responses of several cell types, which might generate confusion in data interpretation. For example, adipocytes are only 50% of cells found in white adipose tissue, and their high heterogeneity is only just starting to be understood [29]. The use of single-cell methods for the T3 response could provide information on which target genes are regulated by which cell subtypes, as already seen with other hormones [30].

Chromatin occupancy is a crucial criterion in our definition of TR target genes, to eliminate genes most likely regulated indirectly. Obviously, classical chromatin immunoprecipitation only provides a one-dimensional picture of the binding sites, forcing us to choose an interval distance within which we can attribute a T3-sensitive gene to a TRBS. Despite our efforts to estimate the best compromise, we may have missed active TRBS due to this limitation. The growing use of methods considering the two-dimensional conformation of chromatin may overcome these problems [31,32,33].

Previous in vitro studies have identified non-DR4 consensus sequences capable of mediating T3/TR/RXR binding and transactivation, called ER6 (everted repeats with a six-nucleotide spacer) and IR0 (inverted repeats half-site without spacer) [34]. This was previously denied by diverse studies [20,35]. Here, the combined analyses of multiple datasets identified DR4 as the unique consensus motif significantly enriched, which raises some doubts about the physiological relevance of the non-DR4 binding sites for T3-mediated transactivation.

Overall, this global analysis outlines a basic problem that should be addressed in further research: the genome contains more than 70,000 DR4-like sequences, which are putative TRBS. Among these, only a few thousand are actually occupied by TR, according to ChIP-seq analysis. Thousands of genes are proximal to these TRBS, but the transcription of only a few hundred of these is upregulated by T3. This suggests that only a fraction of chromatin-bound TR converts T3 binding to transcriptional activation. In the following, we consider promising approaches to overcome the current limitations of the RNA-seq/ChIP-seq, which should allow us to tackle this research area.

(1) Many TR target genes encode extracellular proteins: growth factors, neurotrophins, etc. Therefore, in a tissue, the cellular response to these factors is difficult to separate from a direct, cell-autonomous response to T3. This has been notably exemplified in the brain, where interaction between neighboring cell types is a common theme [20,36]. In mice, the availability of *Thra* and *Thrb* “floxed” alleles enables us to address this point. TR target genes are expected to be downregulated when the T3 response is selectively blocked in the cell type considered. This provides an additional and useful criterion to define TR target genes [20,37];
(2) Cross-species comparisons among mammals or vertebrates could outline gene regulations that are the most relevant for the conserved function of T3. Only a few attempts have been made in this direction, but the regulation of *Klf9* by TR is clearly conserved between mammals and amphibians [38] and crucial for neuronal maturation [39];
(3) Other genomic assays are required to address the influence of chromatin remodeling on cistrome and define the consequences of TR binding, especially on histone tail modifications [40]. This should help to address the possibility that there are many “silent TRBS”;
(4) The development of high-throughput functional assays is needed to demonstrate the ability of TRBS to mediate T3 transactivation. Recently, a proof of principle has been obtained that the so-called synthetic STARR-seq approach can address this question in vitro [28].

In conclusion, we compiled in a single database of sequencing data on the T3 response and TR occupancy in several tissues. This collection sheds new light and opens up a discussion on several mechanistic aspects of TR signaling including TR-mediated repression, hormonal-dependent chromatin occupancy and the nature of TRE. More importantly, this atlas represents a highly valuable tool that may be useful for the entire thyroid hormone community. It can be used to quickly obtain information on specific genes with regard to T3 regulation or TR binding, or to extract a larger list of genes of interest. For example, the list of direct TR target genes in neurons was recently used to screen the effects of around 300 chemicals on thyroid signaling in order to identify and test in vitro the most active compounds in silico [41]. Thus, this atlas is a quickly accessible, easy handling resource to study the thyroid hormone target genes, their function and potential disruption in mice.

## 4. Materials and Methods

### 4.1. Transcriptome Analysis

Raw data were downloaded from the Gene Expression Omnibus. Their accession numbers are listed in Table 1. Sequence reads were aligned with the GRCm38 (mm10) reference genome using Bowtie with the default setup (Galaxy version 2.2.6.2). Reads with an alignment quality inferior to 10 were eliminated. The number of reads assigned to each gene was calculated using htseqcount (Galaxy version 0.6.1galaxy3). Finally, differential analysis of gene expression was performed with DESeq2 (R package version 1.34.0). Differentially expressed genes (DEG) were defined as genes with a mean average expression > 10 and an adjusted *p*-value < 0.05. No threshold was used for the log_2_ fold-change. Using these parameters, genes upregulated were marked with U, downregulated with D, not sensitive to T3 with N and not expressed with NA. For time-course analyses of the T3 response, each timepoint was compared with the control samples prepared from either hypothyroid or euthyroid mice. Importantly, only genes that were regulated in at least one tissue were kept in the atlas. Gene ontology analysis was carried out using the Gene Ontology Resource (http://geneontology.org) (accessed on 23 June 2022).

### 4.2. Cistrome Analysis

Raw data were downloaded from the Gene Expression Omnibus. Their accession numbers are listed in Table 1. Sequence reads were aligned with the GRCm38 (mm10) reference genome using Bowtie with the default setup (Galaxy version 2.2.6.2). MACS (Galaxy version 2.1.1.20160309.0) was used for peak calling, and peaks with a score inferior to 60 were filtered out. Genes within 30 kb of peaks were called out using GREAT (http://great.stanford.edu/public/html/) (accessed on 23 June 2022). According to our estimations (Figure 2B), this distance maximizes the ratio of T3-responsive genes, without excluding genes that have been well-characterized as TRα1 target genes, such as *Klf9* or *Hr* [42]. Bigwig files were obtained by converting the Bedgraph files from MACS2 with the Wig/BedGraph-to-bigWig converter (Galaxy version 1.1.1). Peaks were visualized by uploading Bigwig files to the Integrative Genome Viewer (2.12.3). The distribution of distances of TRBS around TSS, as well as the distribution of TRBS in the genome, were evaluated using PAVIS (https://manticore.niehs.nih.gov/pavis2/) (accessed on 24 June 2022).

A de novo motif search was performed by submitting filtered peaks to the MEME-ChIP program (MEME Suite v5.4.1 https://meme-suite.org/meme/) (accessed on 24 June 2022) [43] looking for motifs of between 6 and 20 nucleotides. Only motifs with a *p*-value < 0.05 were conserved. To estimate the proportion of TRBS that possesses a DR4, we crossed our datasets with a BED file of the more than 70,000 DR4 present in the genome (Table S2). Table S2 was built using FIMO from the normalized probability matrix produced by MEME-ChIP [44].

## Figures and Tables

**Table 1:** Data included in the atlas.

Organ/Cell Type	RNA-Seq Datasets				ChIP-Seq Datasets	
	Publication	Age	Hypothyroidism	Treatment	TR and Publication	Conditions
**Striatum**	Unpublished GSE210976	8 w	-	T3/T4 unique IP injection 20 (T3) and 200 (T4) μg/mL 12 and 24 h	GS-TRα–adult GABAergic neurons Unpublished GSE210975	8 w Euthyroid
					GS-TRα–P15 GABAergic neurons [20] GSE143933	8 w Euthyroid
**Astrocytes** **(in vitro)**	[13] GSE110372	P3	TH-deprived serum for 24 h	T3 culture medium 10 nM 6 and 24 h	-	
**PC cells** **(in vitro)**	[12] GSE68949	E17.5	TH-deprived serum for 24 h	T3 culture medium 10 nM 24 h	-	
**Heart**	[21] GSE124117	8 w	PTU in food (4 weeks)	T3 daily IP injection 1 ug/g of BW Achieved 72 h	GS-TRα Cardiomyocytes [19] GSE125414	P14 Euthyroid
**Skeletal muscle**	[22] GSE146336	Adult	-	T3 daily SC injection 100 nmol/kg BW 7 days	-	
**BAT**	Unpublished GSE201136	8 w	PTU in food (2 weeks)	T3/T4 unique IP injection 20 (T3) and 200 (T4) μg/mL 3, 6, 12 and 24 h	GS-TRα Brown adipocytes Unpublished GSE201136	8 w PTU and PTU + T3/T4
**WAT**	Unpublished GSE210976	8 w	PTU in food (2 weeks)	T3/T4 unique IP injection 20 (T3) and 200 (T4) μg/mL 6 and 24 h	-	
**Liver**	[18] GSE159648	12–14 w	MMI in drinking water (4 weeks)	T3 in drinking water 5 μg/mL 7 days	TRβ1 [15] GSE65947	Adult PTU and PTU + T3
					Biotin-TRβ1 [23] GSE52613	Adult PTU and PTU + T3
					HA-TRβ1 [18] GSE159648	Adult PTU and PTU + T3

PC: primary cerebrocortical, w: weeks, P: postnatal day, E: embryonic day, PTU: propylthiouracil, MMI: methimazole.

## Data Availability

Publicly available datasets were analyzed in this study. They can be found in the Gene Expression Omnibus (GSE210976, GSE210975, GSE143933, GSE110372, GSE68949, GSE124117, GSE125414, GSE146336, GSE201136, GSE159648, GSE65947 and GSE52613).

## Supplementary Materials

The following supporting information can be downloaded at: https://www.mdpi.com/article/10.3390/ijms231911444/s1.
